# Algal Lectins as Potential HIV Microbicide Candidates

**DOI:** 10.3390/md10071476

**Published:** 2012-07-10

**Authors:** Dana Huskens, Dominique Schols

**Affiliations:** Rega Institute for Medical Research, KU Leuven, Minderbroedersstraat 10, B-3000 Leuven, Belgium; Email: Dominique.Schols@rega.kuleuven.be

**Keywords:** algae, lectin, carbohydrate-binding agents, HIV, virus entry, gp120 envelope, microbicide

## Abstract

The development and use of topical microbicides potentially offers an additional strategy to reduce the spread of the Human Immunodeficiency Virus (HIV). Carbohydrate-binding agents (CBAs) that show specificity for high mannose carbohydrates on the surface of the heavily glycosylated envelope of HIV are endowed with potent anti-HIV activity. In fact, a number of algal lectins such as cyanovirin-N, microvirin, microcystis viridis lectin, scytovirin, *Oscillatoria agardhii* agglutinin and griffithsin are considered as potential microbicide candidates to prevent the sexual transmission of HIV through topical applications. They not only inhibit infection of cells by cell-free virus but they can also efficiently prevent virus transmission from virus-infected cells to uninfected CD4^+^ target T-lymphocytes and DC-SIGN-directed capture of HIV-1 and transmission to CD4^+^ T lymphocytes. This review focuses on the structural properties and carbohydrate specificity of these algal lectins, their antiviral activity against HIV and several other enveloped viruses, their safety profile and viral resistance patterns.

## 1. Introduction

Since the discovery of the Human Immunodeficiency Virus (HIV) almost 30 years ago, more than 25 million people have been killed by this virus and approximately 34 million people are estimated to live with HIV. The HIV epidemic continues largely unabated with approximately 8000 new infections every day. The existing HIV drug treatments can control, but are not able to cure HIV infection. The most effective approach to halt the epidemic will be establishing effective prevention methods. This should be a multifaceted approach incorporating multiple types of intervention including behavioral modification, voluntary counseling and HIV testing, condom use, male circumcision, diagnosis and treatment of STDs, vaccination, oral pre- and post-exposure prophylaxis and development of anti-HIV microbicides. A vaccine against HIV should offer the best chance of reducing HIV infection and viral transmission, however, approaches to elicit protective immune responses remain still elusive. Pre-exposure prophylaxis with orally administered anti-retroviral drugs to protect HIV-negative persons may prove to be effective, but there are substantial concerns for toxicities associated with long-term exposure and the risk for selecting resistant virus variants. Topically delivered drugs that can be applied vaginally or rectally, designated microbicides, offer distinct advantages because they would limit the toxicities associated with systemic prophylaxis. An enormous advantage of microbicides is that women can use them without their partners knowing. Although condoms can provide excellent protection against HIV and other sexually transmitted diseases, women are not always able to negotiate condom use. Frequently, men can be reluctant to use condoms with regular partners because it implies that one, or both partners are unfaithful [1]. One study estimated that a single microbicide with only 60% efficacy could prevent over one million new infections per year [2]. However, significant protection has yet to be achieved by this approach and up till now, only one antiviral compound was claimed to be active in a microbicidal gel. The CAPRISA 004 trial investigated a gel containing 1% tenofovir and this gel reduced women’s risk of HIV infection by 39% [3]. In this study participants were counseled to apply no more than two gel doses in 24 h; the first dose within 12 h before sex and second dose as soon as possible but within 12 h after sex (BAT24 dosing regimen). In the VOICE study, however, a large phase IIb clinical trial that investigated tenofovir gel used in a once-daily formulation, unfortunately, no protective effect was observed. In the last decade, there has been a shift from the development of broad-spectrum microbicide products with relative non-specific mechanisms of antiviral action, such as surfactants, to antiretroviral microbicides that target specific steps in the viral life cycle, such as the HIV entry process. 

Infection of CD4^+^ target cells by HIV is a complex, multi-stage process involving viral attachment to host cells and subsequent membrane fusion. HIV enters cells via an interaction of gp120 with the cellular receptor CD4 and a so-called HIV co-receptor (CCR5 or CXCR4), insertion of gp41 into the cellular membrane and membrane fusion. The external gp120 and the transmembrane gp41 are heavily glycosylated proteins and they are present on virion surfaces as trimers of gp120/gp41 complexes [4,5,6]. Approximately half of the total molecular mass of gp120 is contributed by *N*-linked glycans with a small and variable contribution of *O*-linked glycans [7,8]. *N*-linked glycans can be attached to the protein backbone at positions predetermined by short amino acid motifs (N-X-S/T) designated as potential *N*-glycosylation sites (PNGS). *N*-Linked glycans are added co-translationally to newly synthesized polypeptides in the endoplasmic reticulum (ER) [9]. They are linked to the amide side chain of an asparagine residue present in the sequon NX (T/S), where X can be any amino acid except proline. The *N*-linked glycan is assembled as a high mannose type glycan (designated Glc_3_Man_9_GlcNAc_2_) on a membrane-bound dolichyl pyrophosphate precursor. In the ER, the addition of these glycans on the native peptide plays a pivotal role in protein folding. The correctly folded protein then migrates to the Golgi apparatus, where glycosidases and glycosyltransferases process the glycans by removing (trimming) and adding new sugars, creating hybrid and complex type glycans. However, a number of *N*-glycans on Env are resistant to mannose trimming and retain an oligomannose composition [5,10]. These high mannose type glycans appear to be clustered on the gp120 envelope, resulting in an unusual density of such glycans in the envelope of HIV-1 [11]. This high number of high mannose type glycans is peculiar, because human cells rarely express proteins carrying this type of glycan [12]. In addition, these carbohydrate moieties on gp120 act as “shields” to mask neutralization-sensitive epitopes from recognition by the immune system [6]. However, recently, broad and potent neutralizing antibodies were found that are specific for high-mannose-glycan-dependent epitopes and this supports the hypothesis that glycans are important targets on HIV glycoproteins for broad neutralizing responses *in vivo*, providing an important lead for future directions in developing neutralizing antibody-based anti-HIV vaccines [13,14].

Carbohydrate-binding agents (CBAs) interact with the glycans on the viral envelope of HIV and block viral entry into its target cells. Algal lectins can be considered as CBAs with the most potent anti-HIV activity described so far and they are the subject of this review.

## 2. Origin of Algal Lectins

Lectins are proteins of non-immunoglobulin nature, capable of recognition of and reversible binding to carbohydrate moieties of complex glycocongugates without altering the covalent structure of any of the recognized glycosyl ligands. Lectins can be found across a wide variety of different species in nature including prokaryotes, sea corals, algae, fungi, higher plants, invertebrates and vertebrates and are involved in many biological processes, among them host-pathogen interactions, cell-cell communication, induction of apoptosis, cancer metastasis and differentiation, targeting of cells, as well as recognizing and binding carbohydrates. This review will focus on algal lectins with antiviral activity.

In general, algae can be referred to as plant-like organisms that are usually photosynthetic and aquatic, but do not have true roots, stems, leaves, vascular tissue and have simple reproductive structures. The major groups of algae are Divisions Cyanophyta (blue-green algae), Chlorophyta (green algae), Cryptophyta, Chrysophyta (golden-brown algae), Pyrrophyta (dinoflagellates), Bacillariophyta, Euglenophyta and Rhodophyta (red algae). All members of Division Cyanophyta, the “blue-green algae”, are prokaryotic cells with no organized nucleus and no mitochondria or chloroplasts. Members of all other algal divisions are eukaryotic. In recent years, algal lectins that could inhibit the replication of HIV and many other classes of enveloped viruses by interacting with the carbohydrates present on the viral envelopes, were discovered. 

## 3. Structural Properties and Carbohydrate Specificity of Algal Lectins

**Figure 1:**
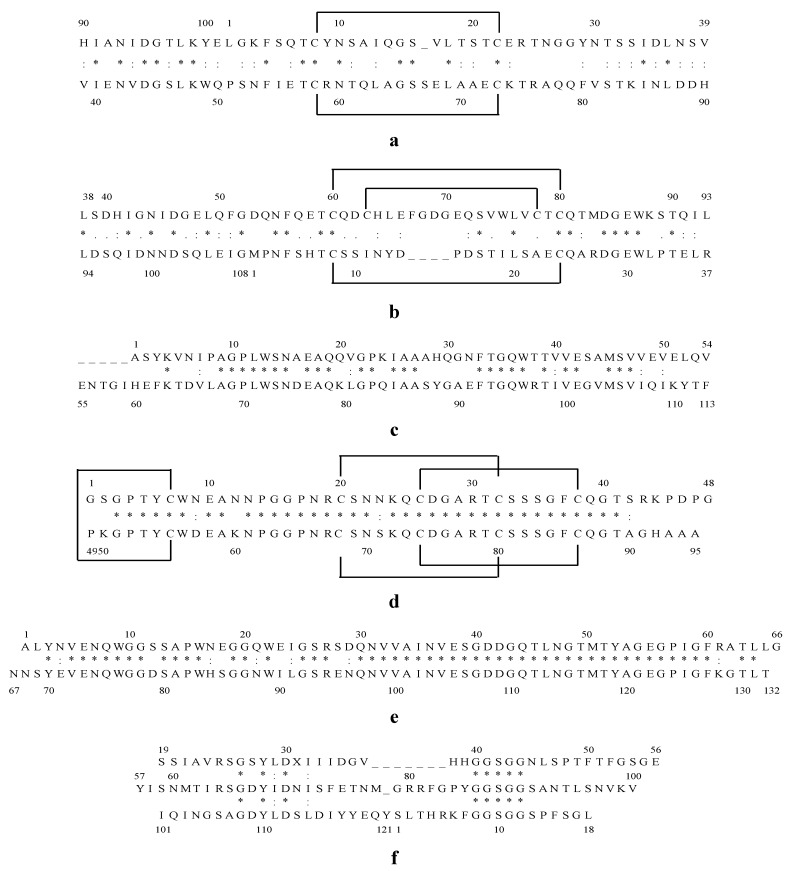
Internal amino acid sequence alignment of Cyanovirin-N (CV-N) (**a**); Microvirin (MVN) (**b**); Microcystis viridis lectin (MVL) (**c**); Scytovirin (SVN) (**d**); *Oscillatoria agardhii* agglutinin (OAA) (**e**); Griffithsin (GRFT) (**f**). Identical residues are indicated by “*” and similar residues by “:”. Disulfide bonds between cysteines are marked with solid lines above the sequence.

**Figure 2:**
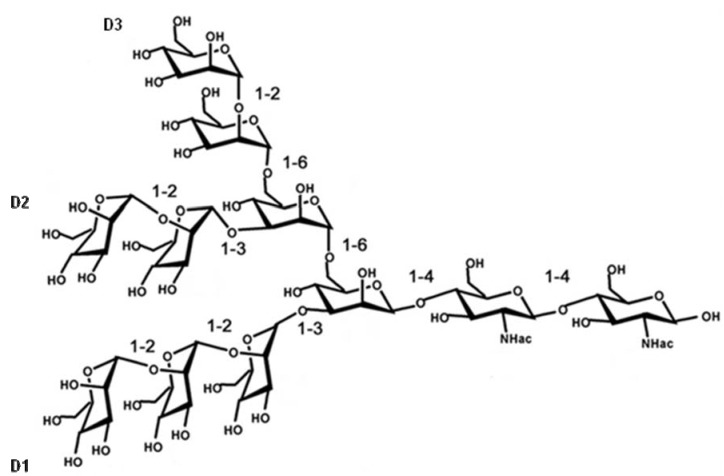
Chemical structure of Man_9_GlcNAc_2_.

Cyanovirin-N (CV-N), an 11 kDa protein derived from the cyanobacterium *Nostoc ellipsosporum*, has been given by far the most attention as antiviral lectin. The primary structure and disulfide bonding pattern of CV-N were determined by conventional biochemical techniques [15,16]. CV-N consists of 101 amino acid residues that can be divided in two internal repeats of 50 and 51 amino acids that show strong sequence similarity to one another, and equivalent positions of the disulfide bonds [16]. Domain A is formed by residues 90–101 and 1–39 and is stabilized by a disulfide bond between Cys-8 and Cys-22 and domain B consists of residues 39–90, with one disulfide bond between Cys-58 and Cys-73 (Figure 1a) [17]. The predominant form of CV-N in solution is the monomeric form and CV-N contains two carbohydrate recognition sites on symmetrically opposed domains of the protein, so it can **cross**-link branched oligomannosides to form higher order structures [17,18,19,20]. CV-N specifically recognizes Manα(1–2)Man linked mannose substructures in the D1 and D3 arms of Man-9 (Figure 2) [18,21].

Microvirin (MVN) is a recently discovered novel lectin isolated from the cyanobacterium *Mycrocystis aeruginosa* and shows 33% identity at the amino acid level with CV-N [22]. MVN has an average molecular mass of 12.7 kDa and consists of 108 amino acids. The amino acid sequence of MVN contains two tandem repeats (residues 1–54 and 55–108) that are 35% identical in sequence, and its three-dimensional structure exhibits a CV-N family fold [17,23]. MVN is monomeric in solution and the structure of MVN includes two homologous domains: domain A is formed by residues 38–93 and is stabilized by two disulfide bonds between Cys-63 and Cys-78 and between Cys-60 and Cys-80, and domain B consists of residues 1–37 and 94–108, with one disulfide bond between Cys-8 and Cys-24 (Figure 1b) [23]. MVN contains only one carbohydrate recognition site, and it shows specificity for Manα(1–2)Man, the disaccharide unit that terminates the arms of high mannose *N*-linked oligosaccharides (Figure 2) [23]. 

Microcystis viridis lectin (MVL) was isolated from the cyanobacterium *Microcystis viridis*. MVL has a molecular weight of 12.2 kDa and consists of 113 amino acids (Figure 1c). The amino acid sequence of MVL contains two highly homologous domains of 54 amino acids with 50% of the amino acids identical between the domains [24]. MVL is a homodimer stabilized by an extensive intermolecular interface between monomers [25]. The specificity of MVL is unique in that its minimal target comprises the Manα(1–6)Manβ(1–4)GlcNAcβ(1–4)GlcNAc tetrasaccharide core of oligomannosides (Figure 2) and Man_3_GlcNAc_2_ binds to a preformed cleft at the distal end of each domain of MVL in a manner that a total of four independent carbohydrate molecules associate with each homodimer [25].

Scytovirin (SVN) is a 9.71 kDa algal lectin isolated from aqueous extracts of the cultured cyanobacterium *Scytonema varium* [26]. This unique protein consists of a single 95 amino acid chain with a highly conserved internal repeat: residues 3–42 and residues 51–90 are 90% identical (36 out of 40), with the remaining three maintaining a similar character and only one significantly different (Figure 1d) [26,27]. The amino acid sequence of SVN contains 10 cysteines forming five disulfide bonds: Cys20–Cys32, Cys26–Cys38, Cys68–Cys80, Cys74–Cys86, and Cys7–Cys55 [28]. SVN is strictly monomeric, with no indication of oligomerization under any conditions [27] and this lectin binds to a specific tetrasaccharide substructure of the high mannose oligosaccharide, the Manα(1–2)Manα(1–6)Manα(1–6)Man tetrasaccharide [29,30]. 

The cyanobacterial lectin *Oscillatoria agardhii* agglutinin (OAA) was isolated from *Oscillatoria agardhii* strain NIES-204 and has a molecular weight of 13.9 kDa [31,32]. The amino acid sequence of OAA consists of 132 amino acids forming two homologous domains, consisting of the residues 1–66 and 67–132, with 75% sequence identity between them (Figure 1e) [31,32]. Sato *et al.* reported that OAA recognizes a long carbohydrate sequence from the nonreducing terminal mannose to the reducing terminal GlcNAc residue with the minimal length of a pentasaccharide, Manα(1–3)Manα(1–6)Manβ(1–4)GlcNAcβ(1–4)GlcNAc (Figure 2) [31]. However, Koharudin *et al.* published that OAA preferentially binds to Manα(1–6)Man and they observed no interaction between the protein and the GlcNAcβ(1–4)GlcNAc disaccharide [33]. In fact, the protein contains two carbohydrate binding sites, positioned symmetrically at two ends, and this group reported that the binding cleft of the protein is too short to accommodate a tetrasaccharide [33,34]. Therefore, they assume that OAA recognizes mainly either of the two Manα(1–6)Man disaccharide units, imbedded within the pentasaccharide glycan [33].

The previously described algal lectins were all isolated from cyanobacteria. However, there is also one lectin described that was isolated from a red alga. Griffithsin (GRFT) was isolated from *Griffithsia* sp. and collected from the waters of New Zealand [35]. GRFT has a molecular weight of 12.7 kDa and a sequence of 121 amino acids (Figure 1f) [35,36]. The residue on position 31 does not match any of the 20 standard amino acids and its functional role is indistinct [35]. GRFT exists exclusively as a 25 kDa dimer and has a domain-swapped structure in which two β-strands of one monomer combine with 10 β-strands of the other monomer to form a β prism of three four-stranded sheets [36,37]. The homodimer has six carbohydrate binding pockets, 3 located at each of the opposite ends of the double-prism homodimer. GRFT binds oligomannose glycans, targeting terminal mannose residues found on Man5–9-GlcNAc_2_ [38].

## 4. Broad Spectrum Anti-HIV Activity of Algal Lectins

The algal lectins described above possess anti-HIV-1 activity, however, for some limited data are available while others were extensively studied. Our main goal here is to give a summary of their anti-HIV activity profile and compare their potency (Table 1). OAA was first described by Sato *et al.* [32] and since then only one study evaluated its antiviral activity [31]. OAA was active in MT-4 cells against the X4 HIV-1 strain IIIB with an EC_50_ of 44.5 nM (Table 1). We recently tested it in PBMC against the HIV-1 X4 and R5 laboratory strains NL4.3 and BaL with mean IC_50_ values of 22 nM [39].

**Table 1:** Broad spectrum anti-HIV activity of algal lectins evaluated in different assay systems.

Algal Lectin	Assay	EC_50_ or IC_50_	Reference
CV-N	HIV-1 X4 laboratory strain in CEM-SS cells	0.1–4.8 nM	[15,40]
	HIV-1 X4 and X4/R5 laboratory strain in CEM cells	0.7–5 nM	[41]
	HIV-1 X4 laboratory strain in MT-4 cells	4 ng/mL	[42]
		16 nM	[43]
	HIV-1 X4 laboratory strain in MT-2 cells	0.4–5.8 nM	[15,40]
	HIV-2 X4 laboratory strain in CEM-SS cells	2.3–7.6 nM	[15,40]
	HIV-2 X4 laboratory strain in CEM cells	2 nM	[41]
	HIV-1 X4 and R5 laboratory strains in PBMC and macrophages	14–160 nM	[41]
	HIV-1 X4 and R5 primary isolate in PBMC and macrophages	0.3–160 nM	[15,40,41,44]
	HIV-2 X4 laboratory strain in PBMC	33 nM	[41]
	Env-pseudotyped X4, R5 and X4/R5 HIV1 strains in TZM-bl cells	0.1–2 nM	[23]
	Env-pseudotyped HIV-1 isolates of clades A/B/C in TZM-bl cells	0.4–18 nM	[44]
	SIV in CEM × 174 cells, MT-4 cells or PBMC	11–160 nM	[15,41]
MVN	HIV-1 X4 laboratory strain in MT-4 cells	6 nM	[43]
	HIV-2 laboratory strain in MT-4 cells	>262 nM	[45]
	HIV-1 X4 and R5 laboratory strains in PBMC	8–22 nM	[43]
	HIV-1 clinical isolates (group M) in PBMC	2–167 nM	[43]
	HIV-1 clinical isolates (group O) in PBMC	>350 nM	[43]
	HIV-2 clinical isolate in PBMC	>350 nM	[43]
	Env-pseudotyped X4, R5 and X4/R5 HIV-1 strains in TZM-bl cells	2–12 nM	[23]
MVL	HIV-1 X4 and R5 Env-mediated fusion in a quantitative vaccinia virus reporter gene assay	30–37 nM	[46]
SVN	HIV-1 X4 laboratory strain in CEM-SS cells	0.3–7 nM	[26,47]
	HIV-1 X4 and R5 primary isolate in PBMC or macrophages	0.4–393.5 nM	[26,44]
	Env-pseudotyped HIV-1 isolates of clades A/B/C in TZM-bl cells	6.2–187 nM	[44]
OAA	HIV-1 X4 laboratory strain in MT-4 cells	44.5 nM	[31]
GRFT	HIV-1 X4 laboratory strain in CEM-SS cells	0.04 nM	[35]
	HIV-1 X4 laboratory strain in MT-4 cells	0.1–0.21 nM	[48,49]
	HIV-1 R5 and X4 strains in MAGI cells	0.03–0.15 nM	[50]
	HIV-2 laboratory strain in MT-4 cells	0.11–0.24 nM	[45]
	HIV-1 X4 and R5 laboratory strains in PBMC	0.16–0.28 nM	[49,50]
	HIV-1 X4 and R5 primary isolate in PBMC or macrophages	0.05–47.6 nM	[35,44,49,50]
	Env-pseudotyped HIV-1 R5 strains in TZM-bl cells	0.02–0.04 nM	[50]
	Env-pseudotyped HIV-1 isolates of clades A/B/C in TZM-bl cells	<3–150 ng/mL	[51,52]
		0.1–56 nM	[44]
	SIV and SHIV in CEM × 174 cells	0.95–1.24 nM	[48]
	SHIV and R5 HIV-1 in PBMC	0.02–0.04 nM	[48]
	SHIV in MOLTCCR5 cells	0.83 nM	[48]

EC_50_ or IC_50_: concentration required to inhibit virus replication by 50%.

Also, the anti-HIV activity of MVL was only tested by one research group and it was found active but not that potent in a quantitative vaccinia virus reporter gene assay (EC_50_ = 30–37 nM). The antiviral activity of several other lectins was investigated more extensively against HIV and Simian-Human Immunodeficiency Virus (SHIV) or Simian Immunodeficiency Virus (SIV) laboratory strains and clinical isolates in several cell lines, PBMC and macrophages as well as their activity against env-pseudotyped HIV isolates in luciferase reporter gene assays in TZM-bl cells based on single-round infections (Table 1). CV-N was active against HIV-1 and HIV-2 laboratory strains with EC_50_ values between 0.1 and 160 nM. CV-N was also tested against clinical isolates from many different clades and was found active with EC_50_ values between 0.3 and 160 nM and CV-N was found active against SIV (Table 1). MVN was active against HIV-1 laboratory strains and clinical isolates with EC_50_ values between 6 and 22 nM and 2 and 167 nM. However, in contrast to CV-N, MVN was not active against a HIV-1 clinical isolate of group O and no activity was found against a HIV-2 laboratory strain and clinical isolate (EC_50_ > 350 nM) (Table 1). SVN was active against HIV-1 laboratory strains and clinical isolates with EC_50_ values between 0.3 and 7 nM and 0.4 and 394 nM, respectively (Table 1) and no data were found on its activity against HIV-2 and SIV. GRFT is the most potent anti-HIV algal lectin described so far and it was active against HIV-1 and HIV-2 laboratory strains with EC_50_ values between 0.03 and 0.28 nM. GRFT showed also potent antiviral activity against different HIV-1 clade clinical isolates with EC_50_ values between 0.05 and 56 nM and it was found active in SIV and SHIV replication assays (Table 1). 

## 5. Algal Lectins as Potential HIV Microbicide Candidates

In order to be efficacious, microbicides must overcome several challenges imposed by the mucosal microenvironment they intend to protect. The complete mechanism of HIV-1 transmission in the female genital tract is not fully understood, but multiple pathways are proposed. Initially, HIV-1 infected donor cells or free virions are trapped in cervical mucus. Free virions may penetrate into thin gaps between squamous epithelial cells of the cervicovaginal mucosa [53]. Once in the epithelium, HIV-1 can encounter Langerhans cells (LCs). Langerhans cells are a subtype of dendritic cells (DCs) that express CD4, CCR5 and the C-type lectin langerin, and these antigen presenting cells form a tight network in the mucosal squamous epithelium. Their cellular processes can reach up to the most superficial layers of the epithelial surface, enabling HIV-1 to directly bind to LCs and become internalized into cytoplasmic vesicles. It is also proposed that HIV-1 infects LCs which migrate to the lymphoid tissues, where HIV-1 is efficiently transmitted to T cells [54]. In addition, CD4^+^ T cells, infiltrated in the vaginal and ectocervical squamous epithelium, can be infected and the virus may also penetrate several layers from the luminal surface, and reach suprabasal or basal epithelial cells that are susceptible to trancytosis, infection or internalization of virions into endocytic compartments [53]. Free virions and HIV-infected donor cells can also penetrate the vaginal epithelium by migrating through microabrasions that can be induced during the intercourse. Once in the mucosal stroma, HIV-infected donor cells are taken up by lymphatic or venous microvessels and transported to local lymph nodes or into the blood circulation, respectively. Penetrated free virions can make contact with DCs, T cells and macrophages in the submucosa. CD4^+^ T cells are probably the principal cell type infected at the portal of entry [55]. Susceptible CD4^+^ T target cell populations in the vagina, ecto- and endocervix are largely spatially dispersed populations lying just beneath the epithelium, and, to a lesser extent, deeper submucosa. Also, these CD4^+^ T cells outnumber macrophages and dendritic cells [55]. Stromal DCs express Dendritic Cell-Specific Intercellular adhesion molecule-3-Grabbing Non-integrin (DC-SIGN) and low amounts of CD4, CCR5 and CXCR4. Multiple mechanisms for how DCs augment infection of T cells have been proposed [56]. In classical *trans* infection, DC-SIGN can act as a receptor that efficiently transfers the virus to CD4^+^ T lymphocytes. This can occur across an “infectious synapse”, which is a zone of DC-T-cell contact where HIV-1 itself and the HIV-1 host-cell receptors are concentrated [57,58]. Alternatively, *trans* infection may also occur by HIV-1 associated with DC-derived exosomes [59,60]. Another mechanism involves the transmission of new virions from productively infected DCs across the infectious synapse to T cells [53,56,60,61]. However, clear evidence for any of these described modes for viral transmission from DCs to CD4^+^ T cells in the genital mucosa is still lacking.

**Figure 3:**
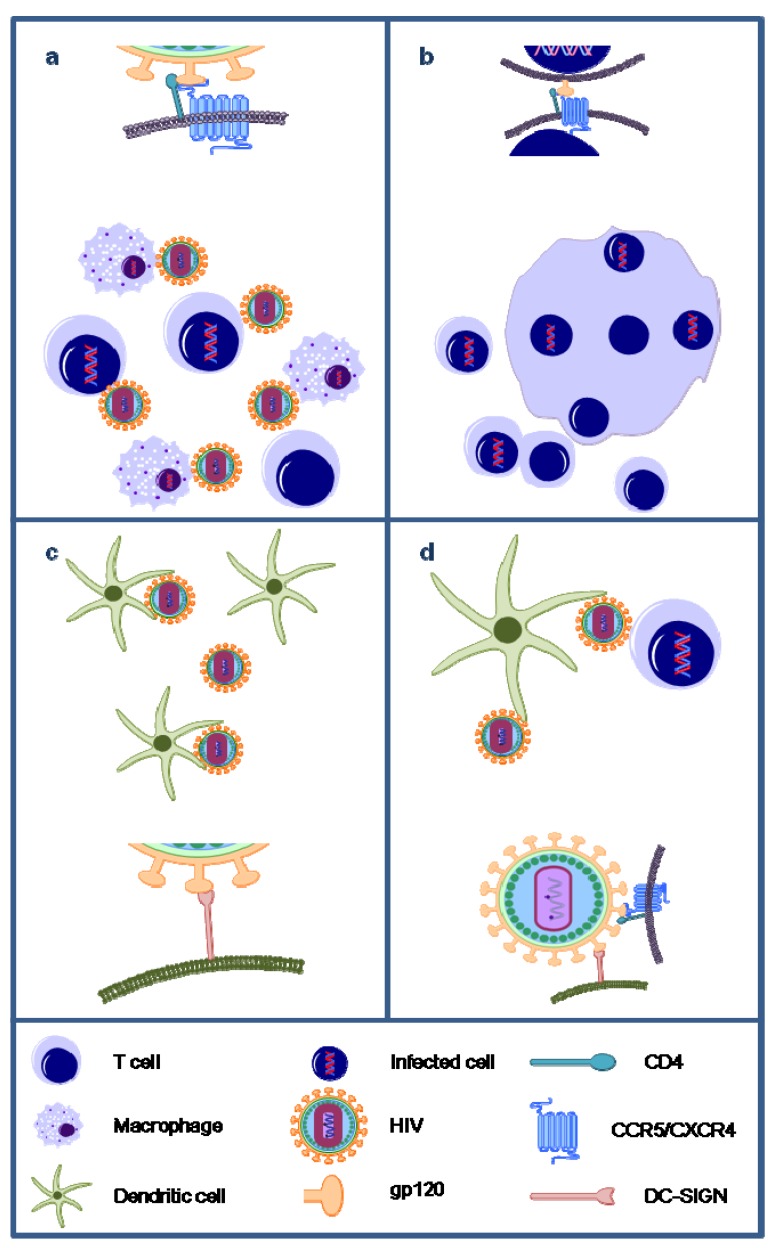
Overview of the unique antiviral activities of carbohydrate-binding agents (CBAs). Algal lectins have been shown to efficiently inhibit the infection of CD4^+^ T cells and macrophages by cell-free HIV particles (**a**); inhibit syncytia formation between HIV-infected cells and uninfected target CD4^+^ T cells (**b**); inhibit the capture of HIV particles by DC-SIGN-expressing cells such as dendritic cells (DCs) (**c**); and inhibit the transmission of DC-SIGN-captured HIV to CD4^+^ target T cells (**d**).

**Figure 4:**
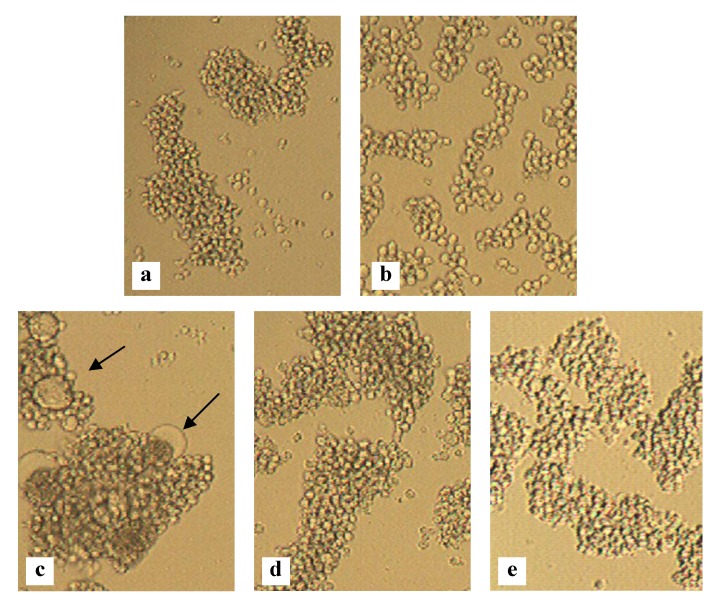
Inhibition of HIV-induced giant cell formation. Light microscopic pictures of the following T cell line cultures: SupT1 cells (**a**); HUT-78 cells persistently infected with HIV-1 IIIB (**b**); Co-culture of SupT1 cells and HUT-78/HIV-1 IIIB cells (several giant cells are indicated with arrows) (**c**); Co-culture of SupT1 cells and HUT-78/HIV-1 IIIB cells in the presence of 140 nM MVN (**d**) or 1 nM GRFT (**e**).

Thus, the ultimate goal is to develop an effective microbicide that not only inhibits the transmission of cell-free viruses (Figure 3a), but also the transmission of donor-HIV-infected T cells (Figure 3b). These HIV-infected cells express the viral glycoproteins on their cell surface and lectins are able to bind and inhibit syncytium formation between infected cells and uninfected CD4^+^ T cells. For the algal lectins CV-N, MVN and GRFT there has been reported that they can inhibit the transmission of HIV between persistently infected cells and uninfected CD4^+^ T cells with IC_50_ values of 4–46 nM, 124 nM and <1 nM, respectively [35,43,62] (Figure 3b and Figure 4). For the algal lectins MVL, SVN and OAA, to our knowledge, no data are available. We tested OAA for its potential to inhibit the transmission of HIV between persistently infected cells and uninfected CD4^+^ T cells and its IC_50_ value was 36 nM [39].

Besides the infection of CD4^+^ T cells and macrophages by cell-free virions and donor-infected cells, DC-SIGN-directed capture of HIV-1 and transmission to CD4^+^ T lymphocytes is considered as an important avenue of primary infection of women exposed to HIV-1 through sexual intercourse [53]. The DC-SIGN receptor binds, just like the lectins, to mannose-rich glycans on the HIV-1 envelope. CV-N, MVN, GRFT and SVN were capable of inhibiting the capture of HIV-1 by DC-SIGN [43,63,64] (Figure 3c). Also, when HIV-1 was already captured by DC-SIGN on Raji/DC-SIGN cells, CV-N, MVN, GRFT and SVN could also inhibit the transmission to CD4^+^ T cells with IC_50_ values between 4 and 69.2 nM, 169 nM, 4.4 and 35 nM and 70.6 and 441.3 nM, respectively [43,63,64] (Figure 3d). For the algal lectins MVL and OAA, to our knowledge, no data are available.

As potential microbicide candidates, CV-N and GRFT were also tested in *ex vivo* cervical explant models and they could potently inhibit infection in these models. Here again, GRFT was more effective at preventing infection than CV-N [51,62]. In these cervical explant tissues there is also a spontaneous migration of CD4^+^ DCs during overnight culture [65] and both CV-N and GRFT could inhibit the transfer of virus from these migratory cells [51,62]. In another preclinical test, CV-N was evaluated in a novel penile tissue explant model [66] and CV-N conferred 95% protection against HIV-1 at 1 µM, which is similar to that seen in cervical explants [66]. 

Finally, CV-N is the only lectin so far tested in *in vivo* transmission models. In a vaginal macaque study, gel solutions that contained 5 mg/mL, 10 mg/mL or 20 mg/mL of CV-N were used to get 85% inhibition of vaginal SHIV challenge [67]. Also, CV-N protected macaques from rectal SHIV challenge [68]. Recently, the activity of *Lactobacillus jensenii* expressing CVN was tested *in vivo* in macaques [69]. The cervicovaginal mucosa in women is coated by a bacterial biofilm including Lactobacillus. This commensal bacterium has a role in maintaining a healthy mucosa and can be genetically engineered to produce antiviral peptides. Lagenaur *et al.* [69] reported a 63% reduction in transmission of a chimeric simian/HIV (SHIVSF162P3) after repeated vaginal challenges of macaques treated with CV-N-expressing *Lactobacillus jensenii*. 

## 6. Activity of Algal Lectins against Other STDs

HIV-1 infection is commonly associated with other sexual transmitted viruses, such as HSV, that facilitate the risk of HIV acquisition and worsen the clinical course of HIV disease [70,71,72]. Therefore, it would be beneficial if a future microbicide was efficient not only against HIV-1, but also against other STDs. Given the high degree of similarity between several enveloped viruses in terms of the presence and role of high-mannose glycans on their envelope glycoproteins, the CBA approach can likely be extended to other enveloped viruses that cause various chronic live-threatening infections in humans. 

Many research groups have investigated the antiviral activity of CV-N and GRFT against different classes of enveloped viruses. CV-N is effective against Ebola virus, influenza A and B, hepatitis C virus, measles virus, herpes simplex virus type-1 (HSV-1) and human herpes virus 6 (HHV-6) [40,62,73,74,75,76,77,78]. In contrast, according to some publications CV-N does not inhibit HSV-1, HSV-2, hepatitis B virus, cytomegalovirus, vaccinia virus and adenovirus type 5 [15,75,78]. In contrast to MVN (IC_50_ > 10 µM), CV-N was active against the enveloped viruses, murine leukemia virus and vesicular stomatitis virus [23].

GRFT can prevent hepatitis C virus infection *in vitro* and mitigate hepatitis C virus infection *in vivo* [79]. Also, GRFT displayed low nanomolar activity against SARS-related coronavirus (SARS-CoV) [36,80]*.* In fact, GRFT was active against coronavirus strains that utilize protein-protein interactions for viral targeting (e.g., ACE2 as a cellular receptor, SARS-CoV, and HCoV-NL63) and those that utilize protein-carbohydrate interactions for viral attachment (*i.e.*, α-2,3-linked sialic acid moieties, IBV-CoV, and HCoV-OC43) [80].

## 7. Safety of Algal Lectins

Irrespective of the potent antiviral activity of these lectins as potential microbicide candidates, safety issues are extremely important and can also contribute to a lack of efficacy of microbicides. The spermicide nonoxynol-9 was the first compound tested profoundly for its potential as an anti-HIV microbicide. It showed *in vitro* activity against HIV-1 and other sexually transmitted infections and prevented SIV infections in macaques [81,82,83,84,85]. However, when nonoxynol-9 was evaluated in clinical trials, it failed to exert protection against HIV-1 transmission in women. Even worse, nonoxynol-9 caused toxic side effects and even enhanced HIV-1 infection and transmission, so that it was no longer pursued as a potential HIV-1 preventive agent [86]. The microbicide cellulose sulfate (CS) was found safe in a phase I safety study [87], however, a phase III clinical trial was interrupted because preliminary results indicated an increased risk of HIV transmission/infection in CS-treated women [88]. So, it is important that microbicidal agents are safe and effective following vaginal or rectal administration, and they should cause minimal, if any, genital side effects by long-term and repeated administration. 

Buffa *et al.* [62] published that CV-N has some mitogenic activity following 3 days exposure to this lectin, and this was associated with a modest increase in expression of gamma interferon, stromal cell-derived factor 1β and interleukin 4. However, 2 h exposure to CV-N had no effect on cytokine expression in PBMC or tissue explant culture over a 24 h period, suggesting that the potential for inflammation is low [62]. In contrast, our group reported that CV-N affected the cell morphology of PBMC and enhanced the expression of the cellular activation markers CD25, CD69 and HLA-DR after 3 days of incubation [41,89]. Also, PBMC activated by CV-N were more susceptible for R5 HIV-1 infection and CV-N exerted a pronounced mitogenic activity and significantly enhanced in PBMC the production of a wide variety of cytokines [41,89].

MVN had, in comparison with CV-N, a much better safety profile. In MT-4 cells and PBMC, MVN did not exert any cellular toxicity at a dose up to 35 µM and 7 µM, respectively, which is in sharp contrast to CV-N (CC_50_ values in MT-4 cells, CEM-SS cells PBMC and TZM-bl cells were 190 nM, 230 nM, 900 nM–1.63 mM and 0.75 mM, respectively) [40,41,43,62]. Also, MVN did not activate PBMC as measured by cellular activation markers but had, however, a substantial effect on PBMC in the release of several pro-inflammatory cytokines [43].

GRFT has no measurable effect on cell viability (CC_50_ > 10 µM in MT-4 cells and PBMC) and does not significantly upregulate described CD markers of T-cell activation [45,48,50]. Also, treatment with GRFT induces only minimal changes in secretion of inflammatory cytokines and chemokines by epithelial cells or human PBMC [50]. In addition, O’Keefe *et al.* [51] evaluated GRFT in the RVI assay (The standard test for safety of vaginal topical products required by the U.S. Food and Drug Administration) and found that GRFT has an acceptable safety profile to proceed to clinical testing. Furthermore, GRFT-P (plant-produced GRFT) was shown to be safe in *ex vivo* human cervical explants [51].

## 8. HIV Resistance

The exposure of HIV to algal lectins will eventually result in resistance. In these resistant viruses an accumulation of amino-acid mutations appears, mainly in the putative *N*-glycosylation motifs of gp120 (either asparagines or serine or threonine), leading to the disruption of the glycosylation site. Currently, limited data are available on the resistance profile of algal lectins. The HIV-1 NL4.3 MVN resistant virus was selected after a long period, 41 cell culture passages, and two pure amino acid mutations (amino acid present in the wild-type virus completely disappeared) were detected that deleted the glycans on position N295 and N392. In addition, two mixed mutations (mixture of original and mutated amino acids) were observed that affected the glycans at position N339 and N386 [43]. In comparison, the HIV-1 NL4.3 CV-N resistant virus was selected after 60 cell culture passages, with two pure mutations that deleted the glycans at positions N339 and N386 [41]. When five independent CV-N-exposed HIV-1 strains were selected, a total of eight different amino acid mutations exclusively located at *N*-glycosylation sites in the envelope surface gp120 were observed [41]. Six of the eight mutations resulted in the deletion of high-mannose type *N*-glycans (*i.e.*, at amino acid positions 230, 332, 339, 386, 392, and 448). Two mutations (*i.e.*, at position 136 and 160) deleted a complex type *N*-glycan in the variable V1/V2 domain of gp120 [41]. Interestingly, the single virus strain that was selected in the presence of CV-N by Witvrouw *et al.* [42] showed glycan deletions at *N* positions 332, 392, 397, 406, and 448. The deletions at 392, 397, and 406 were due to a deletion of a 13-amino-acid stretch 394-TWFNSTWSTEGSN-406 affecting 3 glycosylation sites at the same time. Hu *et al.* [90], assessed the specificity and minimal requirements of deglycosylation for CV-N resistance and indicated that 3–5 high-mannose residues from 289 to 448 on gp120 were correlated with the resistance levels. A single deglycosylation at N295 or N448 by site-directed mutagenesis in a range of primary and T-cell-line-adapted HIV-1 isolates resulted in marked resistance to GRFT but maintained the sensitivity to CV-N [91]. In addition, concomitant lack of glycans at positions 234 and 295 resulted in natural resistance to CV-N, GRFT and SVN, which was confirmed by site-directed mutagenesis [44]. 

Viral drug resistance can be a problem when using a microbicide and thus a high genetic barrier should favor a microbicide candidate. However, the deletion of part of the glycan shield of HIV may also have its benefits. It has been shown that the presence of glycans on the envelope of HIV is of crucial importance of the evasion of the immunological surveillance of the host [92,93,94,95,96,97,98]. CBAs may therefore have a dual mechanism of action. Firstly, they can have direct antiviral activity, by binding to the glycans of the viral envelope. Secondly, their antiviral action can be indirect, resulting from the progressive creation of deletions in the envelope glycan shield, thereby triggering the immune system to act against previously hidden immunogenic epitopes of the viral envelope [99]. 

## 9. Conclusions

The algal lectins CV-N, MVN, GRFT, MVL, SVN and OAA are promising candidate microbicides for the prevention of HIV transmission by interacting with the glycans on HIV gp120. However, these lectins do have unique properties, including the number of carbohydrate recognition sites and their specificity for oligosaccharides. These differences may account for the differences in antiviral activity. Overall, the algal lectins have a broad activity, however, GRFT exhibits superior anti-HIV activity and OAA and MVL were the least active. CV-N and GRFT were studied extensively and showed also antiviral activity against other enveloped viruses. This benefits their usage as microbicides because HIV-1 infection is commonly associated with other sexually transmitted viruses, such as HSV and HCV, that facilitate the risk of HIV acquisition and worsen the clinical course of HIV disease [70,71,72].

Irrespective of the potent antiviral activity of a microbicide candidate, safety issues are extremely important and can also contribute to a lack of efficacy. The use of CV-N as a safe microbicide raises questions because CV-N has clearly stimulatory/mitogenic activity and induces high amounts of a large number of cytokines. In contrast, GRFT has no stimulatory properties and together with its broad and potent antiviral activity, this algal lectin stands out as potential candidate for microbicidal development. Recently, Férir *et al.* [49] combined GRFT with the nucleotide reverse transcriptase inhibitor tenofovir, the CCR5 HIV co-receptor antagonist maraviroc and the gp41 fusion inhibitor enfuvirtide and all combinations were synergistic against HIV-1 clade B and clade C isolates in PBMCs and in CD4^+^ MT-4 cells.

Antiviral activity and safety are important, however, this does not guarantee a successful microbicide. If microbicides have to fulfill their high expectations, they also have to be acceptable, easy to use and affordable [100]. It is however a misconception that proteins, such as the algal lectins, are too expensive for use. For example, GRFT was produced in multigram quantities after extraction from *Nicotiana benthamiana* plants transducted with a tobacco mosaic virus vector expressing GRFT [51]. Also, the use of *Lactobacillus jensenii* expressing a lectin (e.g., CV-N) can reduce the costs of the development of a microbicide and by transforming the vaginal microflora into a “live” bioshield this kind of microbicidal application may be more user-friendly [69]. 

Hopefully, these lectins can contribute to the development of an efficient, safe and affordable microbicide. 
